# Sedoheptulose-1,7-Bisphosphatase is Involved in Methyl Jasmonate- and Dark-Induced Leaf Senescence in Tomato Plants

**DOI:** 10.3390/ijms19113673

**Published:** 2018-11-20

**Authors:** Fei Ding, Meiling Wang, Shuoxin Zhang

**Affiliations:** College of Forestry, Northwest A&F University, Yangling, Shaanxi 712100, China; fding@nwafu.edu.cn

**Keywords:** jasmonates, SBPase, senescence, photosynthesis, chlorophyll, tomato

## Abstract

Leaf senescence represents the final stage of leaf development and is regulated by diverse internal and environmental factors. Jasmonates (JAs) have been demonstrated to induce leaf senescence in several species; however, the mechanisms of JA-induced leaf senescence remain largely unknown in tomato plants (*Solanum lycopersicum*). In the present study, we tested the hypothesis that sedoheptulose-1,7-bisphosphatase (SBPase), an enzyme functioning in the photosynthetic carbon fixation in the Calvin–Benson cycle, was involved in methyl jasmonate (MeJA)- and dark-induced leaf senescence in tomato plants. We found that MeJA and dark induced senescence in detached tomato leaves and concomitantly downregulated the expression of *SlSBPASE* and reduced SBPase activity. Furthermore, CRISPR/Cas9 (clustered regularly interspaced short palindromic repeats (CRISPR)/CRISPR-associated protein 9)-mediated mutagenesis of *SlSBPASE* led to senescence-associated characteristics in *slsbpase* mutant plants, including loss of chlorophyll, repressed photosynthesis, increased membrane ion leakage, and enhanced transcript abundance of senescence-associated genes. Collectively, our data suggest that repression of SBPase by MeJA and dark treatment plays a role in JA- and dark-induced leaf senescence.

## 1. Introduction

Leaf senescence represents the final stage of leaf development and is a crucial phase of plant life cycle. Leaf senescence leads to the remobilization of nutrients and energy to younger developing tissues and storage organs, which ensures offspring production and better survival in plants under certain circumstances [1,2,3]. Under optimal growth conditions, leaf senescence occurs dependent on developmental age. However, unfavorable environmental factors, such as drought, dark, extreme temperatures, salinity, nutrient deficiency, heavy metal toxicity, and pathogens, trigger premature leaf senescence [2,4]. In addition to environmental cues, internal factors, including phytohormones and metabolites, also induce leaf senescence [2,5,6,7]. Leaf senescence is generally featured by rapid degradation of chlorophyll, suppression of photosynthetic capacity and increased expression of senescence-related genes, including *SENESCENCE*-*ASSOCIATED GENE12* (*SAG12*) and *SENESCENCE4* (*SEN4*) [8,9,10,11].

Jasmonates (JAs) are a group of lipid-derived plant hormones, consisting of jasmonic acid and other oxylipin derivatives, such as methyl jasmonate (MeJA) and jasmonoyl-isoleucine (JA-Ile) [12,13]. JAs have been identified as important regulators of various plant developmental processes, including root elongation, seed germination, cell cycle progression and pollen development [12,14,15]. JAs have also been involved in responses to abiotic stresses, such as drought, salinity, heat, and heavy metals [16,17,18,19]. Additionally, JAs play key roles in defenses against pathogen attack, mechanical wounding, and insect wounding [20,21]. JA signal is first perceived by its receptor CORONATINE INSENSITIVE1 (COI1) [22,23,24]. The F-box protein COI1, together with SKP1 and CULLIN1, assembles SKP1-Cullin-F-box complex (SCF^COI1^), which interacts with JASMONATE ZIM DOMAIN (JAZ) family proteins in a JA-dependent manner [22]. Perception of JA signal leads to ubiquitination of JAZ proteins and subsequent degradation by 26S-proteosome. The degradation of JAZ proteins activates various downstream transcription factors, for instance, the IIIe bHLH (basic helix-loop-helix) transcription factors, MYC2 (myelocytomatosis), MYC3, and MYC4 [25,26,27], the IIId bHLH transcription factors, bHLH03, bHLH13, bHLH14, and bHLH17 [28,29,30], and the R2R3-MYB transcription factors, MYB21 (myeloblastosis) and MYB24 [31].

JA was first reported to trigger senescence in detached oat (*Avena sativa*) leaves [32] and a number of subsequent studies have revealed a role for JA in the induction of leaf senescence and in the regulation of senescence-associated genes in a variety of species, including *Zea mays* [33], *Oryza sativa* [34], and *Arabidopsis thaliana* [30,35]. In Arabidopsis, the bHLH subgroup IIIe transcription factors MYC2, MYC3, and MYC4 are key components in the JA signaling pathway and function in the JA-induced leaf senescence [30]. MYC2 binds to the promoter of *SAG29* and activates expression of *SAG29*, thus inducing leaf senescence. Contrary to the activation function of the bHLH IIIe transcription factors, the bHLH subgroup IIId factors bHLH03, bHLH13, bHLH14, and bHLH17 act as repressors of JA-induced leaf senescence by binding to the promoter of *SAG29* and suppressing the MYC2-activated expression of *SAG29* [30]. Interestingly, a previous study has identified Rubisco activase (RCA) in the Calvin–Benson cycle as a regulator in JA-induced leaf senescence. The expression of RCA is downregulated by JA in a COI1-dependent manner and loss-of-function mutant of RCA shows senescence-associated phenotype [35].

Sedoheptulose-1,7-bisphosphatase (SBPase) is a pivotal enzyme, which catalyzes the dephosphorylation of sedoheptulose-1,7-bisphosphate to sedoheptulose-7-phosphate in the Calvin–Benson cycle. SBPase is important for the regeneration of CO_2_ acceptor molecule ribulose-1,5-bisphosphate (RuBP) and its activity is regulated by a variety of factors, such as pH, Mg^2+^ and light in the cycle [36,37,38]. Accumulating evidence supports an important role for SBPase in the photosynthetic carbon fixation, growth, development, and stress tolerance in plants. Downregulation of SBPase using antisense approach in tobacco plants leads to reductions in photosynthesis and growth, while overexpression of SBPase results in enhanced photosynthetic capacity and growth in tobacco, rice, and tomato [39,40,41,42]. SBPase also confers protection against salinity, high temperature and chilling stress in plants [41,43,44,45]. SBPase, together with RCA, has been demonstrated to be downregulated at the transcript level by JA in a manner that depends on COI1 in Arabidopsis [35]. While evidence supports that RCA is involved in JA-induced leaf senescence, whether SBPase plays a role in this process is still largely unknown.

In the present study, we aimed to uncover the role for SBPase in JA- and dark-induced leaf senescence in tomato plants (*Solanum lycopersicum*). We found that methyl jasmonate (MeJA) and dark treatment induced senescence in detached tomato leaves. MeJA substantially suppressed expression of *SlSBPASE* (Solyc05g052600), the gene encoding SBPase in tomato, and induced expression of representative senescence-related genes. CRISPR/Cas9 (clustered regularly interspaced short palindromic repeats (CRISPR)/CRISPR-associated protein 9)-mediated mutagenesis of *SlSBPASE* in tomato plants led to typical senescence-associated phenotype and increased expression of senescence-related genes, suggestive of the role for SBPase in JA-induced leaf senescence in tomato plants.

## 2. Results

### 2.1. MeJA Induces Senescence in Detached Tomato Leaves

**Figure 1:**
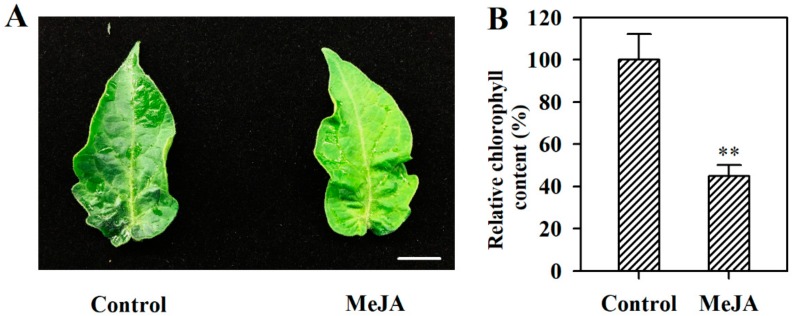
Methyl jasmonate (MeJA) induced tomato leaf senescence. (**A**) Phenotypes of detached tomato leaves treated with MeJA, bar = 1 cm. (**B**) Relative chlorophyll contents of tomato leaves treated with or without MeJA. The chlorophyll content in the leaves without MeJA treatment was set to 100%, and the relative chlorophyll content in the leaves treated with MeJA was calculated accordingly. The values presented are means ± SDs (*n* = 3). Asterisks indicate significant difference at ** *p* < 0.01 between leaves treated with MeJA and control leaves.

To analyze the effect of MeJA on leaf senescence in tomato plants, we treated detached leaves with 100 μM MeJA. Following 4 d treatment, leaves in the control showed no visible changes, while leaves treated with MeJA exhibited leaf yellowing (Figure 1A). Consistent with leaf yellowing, we found that chlorophyll content was reduced by 55% in MeJA-treated leaves compared with that in leaves without MeJA treatment (Figure 1B). These results indicate that 100 μM MeJA is effective in the induction of senescence in tomato leaves.

### 2.2. MeJA Downregulates SlSBPASE, Reduces SBPase Activity, and Suppresses Photosynthesis

**Figure 2:**
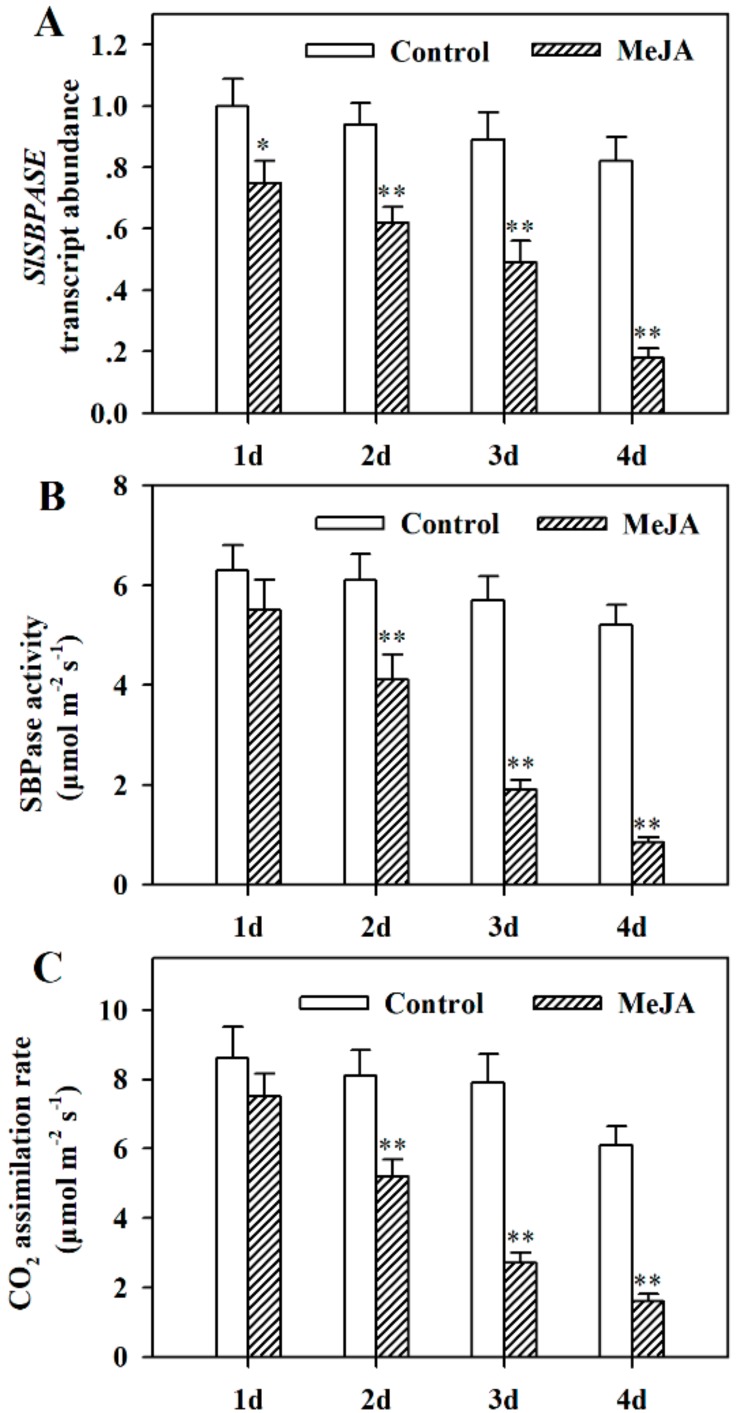
MeJA downregulated *SlSBPASE* expression, reduced sedoheptulose-1,7-bisphosphatase (SBPase) activity, and decreased carbon assimilation rates in detached tomato leaves treated with MeJA for 4 d. (**A**) Analysis of *SlSBPASE* expression level. The expression level in the leaves without MeJA treatment for day 1 was set to 1, and the relative expression levels in the rest of samples were calculated accordingly. (**B**) Quantification of SBPase activity. (**C**) CO_2_ assimilation rates. The values presented are means ± SDs (*n* = 3). Asterisks indicate significant difference at ** *p* < 0.01 between leaves treated with MeJA and control leaves.

To reveal whether SBPase is involved in JA-induced leaf senescence in tomato plants, we examined the transcript abundance of *SlSBPASE*, the gene encoding tomato SBPase, which functions in the carbon fixation, plant growth, and stress tolerance [41,45]. Treatment with 100 μM MeJA considerably downregulated expression of *SlSBPASE*, whereas treatment with water did not significantly change *SlSBPASE* transcript abundance (Figure 2A). In line with *SlSBPASE* transcript abundance were the changes in SBPase activity in MeJA-treated leaves (Figure 2B). Since SBPase is important in the photosynthetic carbon fixation, we next measured the photosynthetic carbon assimilation rate in the control leaves and MeJA-treated leaves. In agreement with reduced *SlSBPASE* transcript abundance and decreased SBPase activity by MeJA, carbon assimilation rates were substantially suppressed in MeJA-treated leaves. These results suggest that MeJA suppresses photosynthesis due, at least partly, to the MeJA-induced downregulation of *SlSBPASE* expression.

### 2.3. MeJA Induces Expression of Senescence-Related Genes

**Figure 3:**
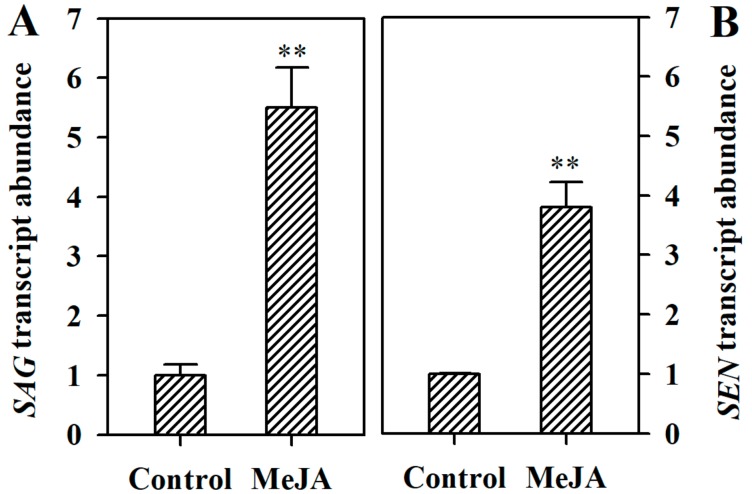
MeJA upregulated the expression of *SAG* (Solyc02g076910.2) and *SEN* (Solyc12g008460.1). (**A**) Transcript abundance of *SAG* in detached tomato leaves treated with or without MeJA for 4 d. (**B**) Transcript abundance of *SEN* in detached tomato leaves treated with or without MeJA for 4 d. The expression level in the leaves without MeJA treatment was set to 1, and the relative expression level in the leaves with MeJA treatment was calculated accordingly. The values presented are means ± SDs (*n* = 3). Asterisks indicate significant difference at ** *p* < 0.01 between leaves treated with MeJA and control leaves.

Senescence marker genes, such as *SAG* (senescence-associated gene) and *SEN* (senescence) are often investigated to assess leaf senescence. To confirm the role for MeJA in the induction of tomato leaf senescence, we measured the transcript abundance of *SAG* (Solyc02g076910.2) and *SEN* (Solyc12g008460.1). We found that the expression of *SAG* and *SEN* was significantly induced in MeJA-treated leaves compared with that in control leaves (Figure 3), supporting the action for MeJA in the induction of tomato leaf senescence.

### 2.4. Dark-Induced Senescence Involves Suppression of SlSBPASE

**Figure 4:**
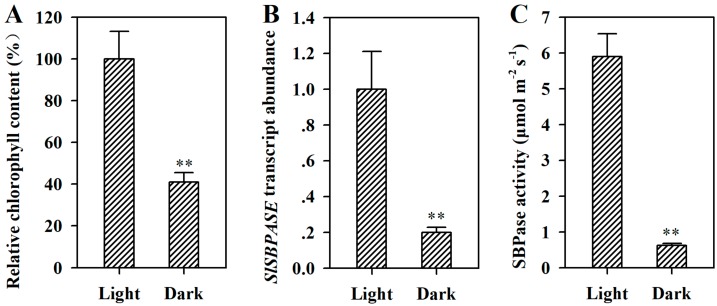
Downregulation of *SlSBPASE* was involved in dark-induced senescence. (**A**) Relative chlorophyll contents of tomato leaves treated with or without dark. The chlorophyll content in the leaves without dark treatment was set to 100%, and the relative chlorophyll content in the leaves treated with dark was calculated accordingly. (**B**) Transcript abundance of *SlSBPASE* in tomato leaves with or without dark treatment. The expression level in the leaves without dark treatment was set to 1, and the relative expression level in the leaves with dark treatment was calculated accordingly. (**C**) SBPase activity in tomato leaves with or without dark treatment. The values presented are means ± SDs (*n* = 3). Asterisks indicate significant difference at ** *p* < 0.01 between leaves treated with dark and control leaves.

Dark is one of the environmental cues that trigger leaf senescence in plants. To investigate whether SBPase is involved in dark-induced leaf senescence, we examined the transcript abundance of *SlSBPASE* in detached leaves incubated in the dark for 4 d. The results showed that the expression of *SlSBPASE* was dramatically inhibited in dark-treated leaves compared with that in control leaves. As SBPase activity requires the activation of light, to measure SBPase activity, we placed detached leaves under illumination of 300 μmol s^−1^ m^−2^ for 10 min following dark treatment. It was observed that SBPase activity was severely suppressed as a consequence of dark treatment. Additionally, as an important physiological marker, chlorophyll content was also measured in detached leaves that had been subjected to dark. We found that dark substantially reduced the level of chlorophyll (Figure 4). These results demonstrate that SBPase may play a role in diverse types of senescence, including JA-induced senescence and dark-induced senescence.

### 2.5. Mutation of SlSBPASE Causes Typical Phenotypes of Senescence

Previously, we have generated *slsbpase* mutant using CRISPR/Cas9-mediated gene editing technology in tomato plants. Briefly, a target sequence (5′-TGCGCCTAAATCATCACTAAAGG-3′) in the second exon of *SLSBPASE* (Solyc05g052600) was selected. A 19-bp sgRNA oligo (5′-GCGCCTAAATCATCACTAA-3′) was cloned into an expression cassette, consisting of chimeric RNA driven by the AtU6 promoter and optimized Cas9 driven by the enhanced CaMV 35S promoter (BIOGLE, Jiangsu, China). *Agrobacterium tumefaciens* harboring the CRISPR/Cas9-*SlSBPASE* plasmid was used to transform tomato plants. Transgenic plants were selected by hygromycin resistance and the genomic DNA from transgenic leaves was used as template to amplify *SlSBPASE* fragment by PCR. The sequencing chromatograms of PCR products were analyzed for *SlSBPASE* mutation. Transgenic plants with homozygous mutation in *SlSBPASE* were used in this study.

**Figure 5:**
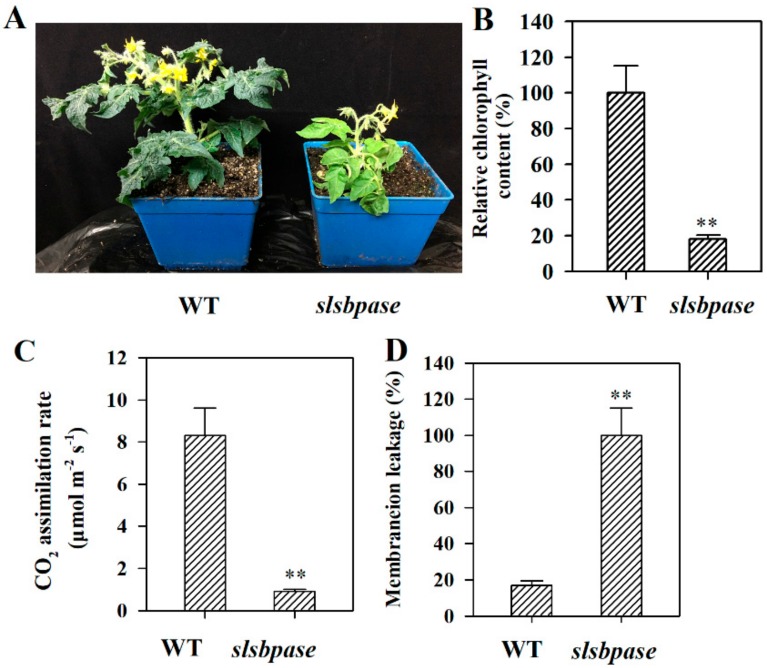
Mutation of *SlSBPASE* led to senescence-associated characteristics in *slsbpase* mutant plants. (**A**) Phenotypes of *slsbpase* mutant plants and their wild type counterparts. (**B**) Relative chlorophyll contents of mutant and wild type leaves. The chlorophyll content in the wild type was set to 100%, and the relative chlorophyll content in the mutant was calculated accordingly. (**C**) CO_2_ assimilation rates in the mutant and wild type plants. (**D**) Membrane ion leakage in the leaves of mutant and wild type plants. The ion leakage in the mutant was set to 100%, and the ion leakage in the wild type was calculated accordingly. The values presented are means ± SDs (*n* = 3). Asterisks indicate significant difference at ** *p* < 0.01 between *slsbpase* mutant plants and their wild type counterparts.

Mutation of *SlSBPASE* led to a phenotype that was quite distinct from wild type plants. The growth was severely restricted in *slsbpase* mutant plants compared with that in wild type plants. *slsbpase* mutant plants developed a leaf chlorosis phenotype (Figure 5A), consistent with the phenotype of senescence. Measurement of chlorophyll content, a physiological marker of senescence, showed that chlorophyll content in *slsbpase* mutant leaves was much lower than that in wild type leaves (Figure 5B). Further analysis demonstrated that mutation in *SlSBPASE* led to a substantial reduction in photosynthesis (Figure 5C). Membrane ion leakage is an important signature of leaf senescence [9], we therefore examined the membrane ion leakage in *slsbpase* mutant plants and their wild type counterparts. The results showed that ion leakage in *slsbpase* mutant plants was dramatically increased compared with that in wild type plants (Figure 5D). These results indicate that SBPase may be involved in the tomato leaf senescence.

### 2.6. Mutation in SlSBPASE Alters Expression of Senescence-Related Genes and Photosynthesis-Related Genes

**Figure 6:**
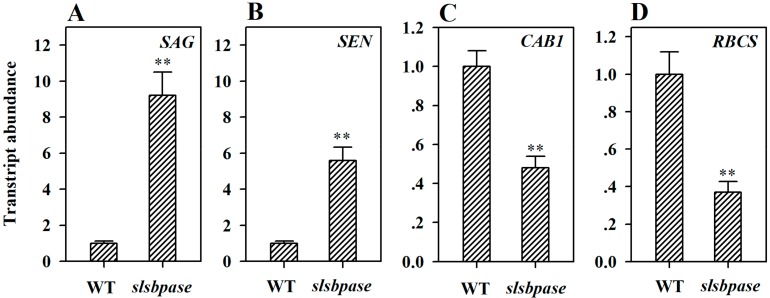
Mutation of *SlSBPASE* upregulated the expression of *SAG* (SENESCENCE-ASSOCIATED GENE, Solyc02g076910.2) and *SEN* (SENESCENCE, Solyc12g008460.1), and downregulated the expression of *CAB1* (chlorophyll a/b-binding protein 1, Solyc02g070970.1) and *RBCS* (Rubisco small subunit, Solyc07g017950.2) in *slsbpase* mutant plants. Transcript abundance of *SAG* (**A**), *SEN* (**B**), *CAB1* (**C**), and *RBCS* (**D**) in the leaves of *slsbpase* mutant and wild type plants. The expression levels in the wild type leaves were set to 1, and the relative expression levels in the mutant leaves were calculated accordingly. The values presented are means ± SDs (*n* = 3). Asterisks indicate significant difference at ** *p* < 0.01 between *slsbpase* mutant plants and their wild type counterparts.

To further reveal the involvement of SBPase in leaf senescence in tomato plants, we compared the expression of senescence-related genes and photosynthetic genes between *slsbpase* mutant plants and their wild type equivalents. The expression of *SAG* and *SEN* was substantially upregulated, while the expression of *CAB1* (chlorophyll a/b-binding protein 1, Solyc02g070970.1) and *RBCS* (Rubisco small subunit, Solyc07g017950.2) was significantly downregulated in *slsbpase* mutant plants compared with their wild type counterparts (Figure 6). The induced expression of *SAG* and *SEN* and the decreased expression of *CAB1* and *RBCS* are key features associated with leaf senescence, these results thus suggest a role for SBPase in the senescence of tomato leaves.

## 3. Discussion

Being sessile, plants have to adapt to a plethora of adverse environmental conditions. Plant hormones play crucial roles in responses to abiotic stress factors, such as drought, cold, heat and nutrient deficiency, and biotic stress factors, including pathogens and herbivores. Jasmonates (JAs), consisting of jasmonic acid and its derivatives, are a class of lipid-derived plant hormones important for development and stress responses in plants. Numerous studies have established that JA is involved in developmental processes, such as seed germination, root growth, pollen fertility, and senescence. As early as 1980, JA was reported to induce leaf senescence [32] and since then, progress has been made in the understanding of the role for JA in the induction of leaf senescence. Nevertheless, the molecular mechanisms of JA-induced leaf senescence remained largely unknown until recently [30,35,46]. A large number of studies on the elucidation of JA-induced leaf senescence are confined to the model species *Arabidopsis thaliana* and our knowledge on the role of JA in the induction of leaf senescence in a horticultural crop species, such as *Solanum lycopersicum* (tomato), is rather scanty. In the present work, we investigated the effect of MeJA on the induction of leaf senescence in tomato plants. We were particularly interested to understand how SBPase, an enzyme in the Calvin–Benson cycle, might be involved in the MeJA-induced leaf senescence. We found that exogenous MeJA led to senescence in detached tomato leaves and concomitantly caused severe downregulation of *SlSBPASE*, the gene encoding SBPase in tomato. Thus, JA-repressed expression of SBPase correlated with JA-induced leaf senescence. In addition, knockout of SBPase resulted in typical senescence-related features in *slsbpase* mutant plants. Interestingly, we also found that dark-induced senescence was in concert with repressed expression of *SlSBPASE* and reduced SBPase activity. These results suggest that JA suppression of SBPase plays a role in the JA- and dark-induced senescence in tomato plants.

Previous studies have shown that JA induced leaf senescence in different species, including oat, rice, maize, and Arabidopsis [32,33,34,35,46,47,48,49]. In the present study, it was found that MeJA-treated leaves displayed leaf yellowing and quantification of chlorophyll content revealed that chlorophyll content was dramatically reduced in MeJA-treated leaves compared with that in control leaves. Leaf yellowing and decreased chlorophyll content are generally associated with leaf senescence, it is thus suggested that JA induced the senescence in detached tomato leaves. To further support that JA induced leaf senescence, we measured the transcript abundance of senescence marker genes, including *SAG* (Solyc02g076910.2) and *SEN* (Solyc12g008460.1), in MeJA-treated leaves. The expression of *SAG* and *SEN* was substantially upregulated in MeJA-treated leaves in comparison with that in control leaves. The expression pattern of these marker genes provided further evidence that JA caused the induction of tomato leaf senescence. Correlated with JA-induced leaf senescence, it is interesting to observe that *SlSBPASE*, which encodes SBPase in the Calvin–Benson cycle in tomato, was dramatically downregulated by MeJA. The downregulation of *SBPASE* by MeJA was also observed in Arabidopsis in a previous study [35]. Further analysis showed that in line with decreased transcript abundance of *SlSBPASE*, SBPase activity was obviously reduced in MeJA treated leaves. Given that SBPase is a key enzyme in the photosynthetic carbon fixation in tomato plants [41,45], we expected a significant reduction in the photosynthetic carbon assimilation in MeJA-treated leaves and measurement of carbon assimilation rates confirmed our expectation. The observation that JA-induced leaf senescence concurred with JA repression of SBPase may suggest a role for SBPase in JA-induced leaf senescence. The effects of JA on photosynthesis have been examined extensively in previous studies, which have yielded mixed results [50]. While a preponderance of studies support that JA reduces photosynthesis [50,51], there are studies demonstrating that herbivory-induced JA stimulates photosynthesis [50,52,53]. Our results support that JA suppresses photosynthesis via downregulation of SBPase, providing additional evidence for JA-induced inhibition of photosynthesis.

Furthermore, we observed that the expression of *SlSBPASE* and activity of SBPase were significantly decreased in the dark-induced senescent tomato leaves. This observation implies that the decrease in SBPase is also associated with dark-induced leaf senescence. Thus, it can be drawn that downregulation of SBPase may be involved in both dark- and JA-induced leaf senescence.

To further validate the involvement for SBPase in JA-induced senescence of tomato leaves, we made an investigation of SBPase-knockout mutant plants that we previously generated using CRISPR/Cas9 gene editing technology. We found that mutation in *SlSBPASE* led to diverse senescence-associated features in tomato plants. *slsbpase* mutant plants exhibited leaf chlorosis and contained lower chlorophyll compared with wild type plants. In addition, carbon assimilation rates were largely inhibited by mutation of *SlSBPASE*. Membrane integrity is disrupted as a result of senescence and membrane ion leakage is thus often used to assess leaf senescence and cell death [2,9]. We found that membrane ion leakage was enhanced in *slsbpase* mutant plants compared with their counterparts. Further, it was observed that expression of senescence-induced genes, *SAG* and *SEN*, was induced in tomato mutant leaves, whereas expression of senescence-suppressed genes, *CAB1* and *RBCS*, was significantly reduced in *slsbpase* mutants. These results were consistent with our observation of JA-induced senescence in detached leaves. SBPase has been found to be critical in the accumulation of carbohydrates in tomato plants. A small decrease in SBPase activity severely reduces the accumulation of starch and sucrose [41]. It is thus expected that mutation of *SlSBPASE* led to dramatic decline in the levels of carbohydrate, which may also contribute to the accelerated premature senescence in tomato leaves. It is therefore suggested that JA repression of SBPase plays a role in JA-induced leaf senescence in tomato plants.

In summary, this study has demonstrated that MeJA- and dark-induced suppression of Calvin–Benson cycle enzyme SBPase may be involved in MeJA- and dark-induced senescence in tomato leaves. MeJA triggers leaf senescence and simultaneously downregulates SBPase at both the transcript and protein levels. Mutation of *SlSBPASE* leads to diverse senescence-associated features, including leaf yellowing, loss of chlorophyll, upregulation of senescence-induced genes, and downregulation of senescence-reduced genes. Dark-induced senescence might also involve reduction in SBPase. However, due to limited data, the underlying molecular mechanism of SBPase involvement in JA-induced senescence of tomato leaves remains largely unclear. Future studies on the identification of transcription factors capable of binding to *SlSBPASE* promoter may deepen our understanding of the role for SBPase in JA-induced leaf senescence in tomato plants.

## 4. Materials and Methods

### 4.1. Plant Materials

Tomato (*Solanum lycopersicum* cv. Micro-Tom) seeds of *slsbpase* mutant and wild type plants were germinated at 25 °C in the dark on filter paper in petri dishes. Germinated seeds were grown in peat and vermiculite (3/1, *v*/*v*) in 12 cm × 12 cm × 10 cm pots with the following growth conditions: 400 μmol mol^−1^ of CO_2_, photon flux density of 300 μmol·m^−2^·s^−1^, day/night temperature of 25/20 °C, relative humidity of 60 to 65% and a photoperiod of 16 h.

For analysis of JA-induced leaf senescence, fully expanded leaves were detached from tomato plants at the 6-leaf stage and were put into peri dishes containing either 30 mL water or 30 mL 100 μM MeJA (Sigma-Aldrich, St. Louis, MO, USA). Leaves were floating either on water or MeJA solution. All petri dishes were placed under the same growth conditions as mentioned above for 4 d. For analysis of dark-induced leaf senescence, detached tomato leaves were floating on water in petri dishes, which were then placed in the dark for 4 d.

### 4.2. Measurement of Chlorophyll Content

Following treatment, leaf discs were punched with a cork borer from leaves and 0.1 g samples were incubated in 10 mL 80% acetone (*v*/*v*) in the dark until leaf discs were fully degreened. Then, absorbance at 647 and 664 nm was measured and the chlorophyll contents were calculated.

### 4.3. Measurement of Membrane Ion Leakage

Leaves from *slsbpase* mutant plants and wild type plants were collected and incubated in deionized water with gentle shaking overnight, and the conductivity of the incubated solution was measured as C1. Then, leaves were boiled and the conductivity of the solution was measured as C2. The relative membrane ion leakage is calculated as the ratio of C1:C2.

### 4.4. Measurement of Carbon Assimilation Rates

Carbon assimilation rates were measured using a portable photosynthesis system (LI-6400, LI-COR Biosciences, Lincoln, NE, USA). The system has CO_2_ and H_2_O analyzers in the sensor head. Measurements of photosynthesis are based on the differences in CO_2_ in an air stream through the leaf cuvette. The CO_2_ over the leaf is set to be constant, then the CO_2_ concentration of air leaving the chamber is determined. The measurements were conducted on young fully expanded leaves of *slsbpase* plants and wild type plants.

### 4.5. Determination of SBPase Activity

SBPase activity was measured as described in previous studies [54,55]. Leaf samples (0.1 g) were extracted with 1 mL extraction buffer containing 50 mM Hepes (pH 8.2), 5 mM MgCl_2_, 1 mM EDTA, 1 mM EGTA, 10% glycerol, 2 mM benzamidine, 2 mM amino caproic acid, 0.5 mM phenylmethylsulfonyluoride (PMSF), and 10 mM dithiothreitol (DTT). The homogenate was centrifuged and the supernatant was collected. For the activity assay, 20 μL of each protein sample was added to 80 μL of assay buffer (50 mM Tris, 15 mM MgCl_2_, 1.5 mM EDTA, 10 mM DTT, 2 mM SBP) and incubated at 25 °C for 5 min. The reaction was terminated by addition of 50 μL of 1 M perchloric acid. The samples were then centrifuged for 5 min and the supernatant was assayed for phosphate. Fifty microliters of samples and phosphate standards (0–0.5 mM NaH_2_PO_4_) were incubated with 850 μL molybdate solution (0.3% ammonium molybdate in 0.55 M H_2_SO_4_) for 10 min. One-hundred-and-fifty microliters of malachite green (0.035% malachite green and 0.35% polyvinyl alcohol) was added and the samples incubated for a further 45 min at room temperature before measuring the absorbance at 620 nm.

### 4.6. Measurements of Transcript Abundance of SlSBPASE, SAG, SEN, CAB1, and RBCS

Transcript abundance was measured by quantitative real-time PCR. RNA was extracted from detached leaves of different treatments and was used as template for cDNA synthesis. Quantitative real-time PCR was performed using SYBR^®^ Premix Ex Taq™ according to manufacturer’s instructions (TaKaRa, Dalian, China). Tomato actin gene was used as an internal constitutively expressed control. Each real-time PCR reaction was performed in a 25 µL final volume on an iQ5 Multicolor Real-Time PCR Detection System (BIO-RAD, USA) with the following program: 1 cycle of 30 s at 95 °C, followed by 40 cycles of 5 s at 95 °C, 30 s at 60 °C. The primers for *actin* were ATGTATGTTGCTATTCAGGCTGTG (Forward) and TAACCCTCGTAGATAGGGACAG (Reverse). The primers for *SlSBPASE* were CGTGACATCTCCAACAGCTAAGG (Forward) and CATCGCTGCTGTAACCTCCAG (Reverse). The primers for *SAG* were TGCAGTAGCAGCTATGGAAGG (Forward) and ACACCATCTGCTGCCTGGTAT (Reverse). The primers for *SEN* were AGGGTAGTGGAAATCTTGGAG (Forward) and GTTCCTTCAGCAATTGCTTTA (Reverse). The primers for *CAB1* were AGGATCACTATGAGAAAGGCTG (Forward) and CACCTCAAGTTCACGGTTCTT (Reverse). The primers for *RBCS* were AATGCCAGGCTACTATGATGG (Forward) and ATGCACTGTGCCTGCTTAACA (Reverse).

### 4.7. Statistical Analysis

The values presented are the means ± SDs. Student’s *t*-test was performed to compare the difference between *slsbpase* mutant plants and wild type plants, and between MeJA-treated leaves and control leaves. Asterisks indicate significant difference at *p* < 0.05 or *p* < 0.01.
